# FoodPro: A Web-Based Tool for Evaluating Covariance and Correlation NMR Spectra Associated with Food Processes

**DOI:** 10.3390/metabo6040036

**Published:** 2016-10-19

**Authors:** Eisuke Chikayama, Ryo Yamashina, Keiko Komatsu, Yuuri Tsuboi, Kenji Sakata, Jun Kikuchi, Yasuyo Sekiyama

**Affiliations:** 1 Department of Information Systems, Niigata University of International and Information Studies, 3-1-1 Mizukino, Nishi-ku, Niigata 950-2292, Japan; s13178yr@st.nuis.jp; 2 RIKEN Center for Sustainable Resource Science, 1-7-22 Suehiro-cho, Tsurumi-ku, Yokohama 230-0045, Japan; keiko.komatsu@riken.jp (K.K.); yuuri.tsuboi@riken.jp (Y.T.); kenji.sakata@riken.jp (K.S.); jun.kikuchi@riken.jp (J.K.); 3 Graduate School of Medical Life Science, Yokohama City University, 1-7-29 Suehiro-cho, Tsurumi-ku, Yokohama 230-0045, Japan; 4 Graduate School of Bioagricultural Sciences, Nagoya University, 1 Furo-cho, Chikusa-ku, Nagoya 464-0810, Japan; 5 Food Research Institute, National Agriculture and Food Research Organization (NARO), 2-1-12 Kannondai, Tsukuba 305-8642, Japan; sekiyama@affrc.go.jp

**Keywords:** NMR, web tool, food

## Abstract

Foods from agriculture and fishery products are processed using various technologies. Molecular mixture analysis during food processing has the potential to help us understand the molecular mechanisms involved, thus enabling better cooking of the analyzed foods. To date, there has been no web-based tool focusing on accumulating Nuclear Magnetic Resonance (NMR) spectra from various types of food processing. Therefore, we have developed a novel web-based tool, FoodPro, that includes a food NMR spectrum database and computes covariance and correlation spectra to tasting and hardness. As a result, FoodPro has accumulated 236 aqueous (extracted in D_2_O) and 131 hydrophobic (extracted in CDCl_3_) experimental bench-top 60-MHz NMR spectra, 1753 tastings scored by volunteers, and 139 hardness measurements recorded by a penetrometer, all placed into a core database. The database content was roughly classified into fish and vegetable groups from the viewpoint of different spectrum patterns. FoodPro can query a user food NMR spectrum, search similar NMR spectra with a specified similarity threshold, and then compute estimated tasting and hardness, covariance, and correlation spectra to tasting and hardness. Querying fish spectra exemplified specific covariance spectra to tasting and hardness, giving positive covariance for tasting at 1.31 ppm for lactate and 3.47 ppm for glucose and a positive covariance for hardness at 3.26 ppm for trimethylamine *N*-oxide.

## 1. Introduction

Generally, foods from agriculture and fishery products are processed using various technologies. A given foodstuff is a complex matrix including several compounds, from small molecules to macromolecules and with a wide range of physicochemical properties. Therefore, an exhaustive food characterization needs a versatile methodology capable of characterizing such chemical mixtures. Moreover, the chemical and physical properties of foods, which are associated with biochemical complexity such as molecular crowding or gelatinization, can be altered via various food processing techniques such as storage, aging, cutting, kneading, and cooking [1,2,3]. Foods can be easily affected by chemical reactions caused by heat energy and/or radical, enzymatic, and microbial degradation [4,5,6]. Therefore, molecular mixture analysis during food processing has the potential to help us better understand the involved molecular mechanisms, enabling better cooking of the analyzed food.

As nuclear magnetic resonance (NMR) has great potential to characterize molecular mixtures [7,8], we have focused on realizing its potential for food analysis [9,10,11,12]. NMR-based molecular mixture analysis is now performed at several institutions, and all data collected in a single study are frequently collected on an individual instrument at a single location. According to cross-site analytical validity studies, the interconvertibility of NMR data among different institutions is a distinct advantage of the NMR-based approach [13,14,15]. Such wide data dissemination is essential for industrial applications such as evaluation of agricultural products and food quality control. Therefore, by freely distributing our NMR database over the Internet, we can assist a wide range of potential users.

Although NMR approaches for analyzing harvested raw agriculture or fishery materials have been widely reported by us [16,17,18,19,20] and several other groups [21,22,23], systematic analysis throughout food processing toward a variety of materials is not reported, to the best of our knowledge. Namely, a database focusing on the accumulation of NMR spectra from various food processing sources has not been constructed till date. Therefore, we have started developing such a database using bench-top-type NMR with the intended future aim of in situ characterization at various production sites [24,25,26,27,28,29]. Simultaneously, we have accumulated subjective human data accompanied by physical property data. Therefore, our developed tool aimed to predict similar spectral/physical/subjective properties when querying with a user NMR spectrum.

## 2. Results and Discussion

**Figure 1:**
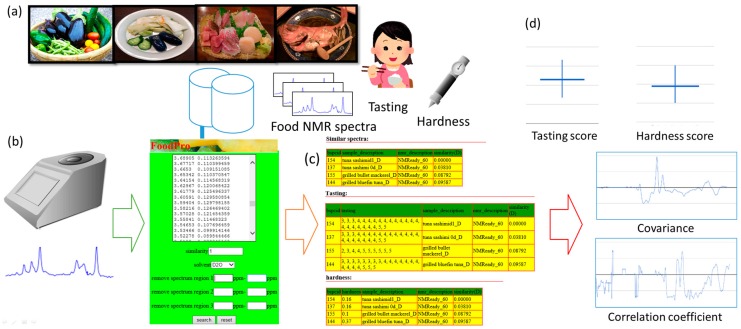
FoodPro summary with screenshots. (**a**) Construction of a database for NMR spectra associated with food processing, tasting, and hardness of which the experimental data were newly measured and accumulated into FoodPro; (**b**) User submits a food ^1^H NMR query on the web portal of FoodPro; FoodPro (**c**) searches similar NMR spectra within a specified similarity threshold with tastings and hardnesses, and (**d**) suggests estimated scores for tasting and hardness based on the experimental tasting and hardness data in the database. Further, FoodPro computes covariance and correlation spectra between tasting (or hardness) and NMR bin intensities along chemical shift.

FoodPro [30] is a novel web-based tool (Figure 1) accumulating 367 experimental food bench-top NMR spectra, 1753 tastings (taste evaluations), and 139 hardnesses into a core database (Figure 1a). The NMR spectra comprise 236 aqueous (extracted in D_2_O) and 131 hydrophobic (extracted in CDCl_3_) spectra. FoodPro affords a food NMR spectrum as a user query (Figure 1b); searches the database; offers similar retrieved NMR spectra, tastings, and hardnesses (Figure 1c); and computes estimated tasting and hardness, covariance, and correlation spectra (Figure 1d). FoodPro is intended to analyze food NMR spectra specifically focusing on aspects such as processing, cooking, storage, and fermentation. By registering NMR spectra, tastings, and hardness experimental values, it can link food NMR spectra with them. For quantifying values of food, data provided by human subjects, such as taste or texture, is important. FoodPro is a web-based tool that can judge similarity between a user query and database NMR spectra. It can analyze relations between a user query and tasting or hardness, which are associated with taste or texture, respectively. System and entity–relationship diagrams are shown for specifying data flow and structure of the database (Figure S1 in Supplementary Materials). Detailed information about this novel tool concerning how a query can be set, the structure of the displayed results, examples of food NMR spectra in the database, a summary of the database and search sensitivity of the tool, and examples of covariance and correlation spectra for fish NMR spectra are described below in that order.

How to submit a query on the FoodPro web interface is described in Figure 1b (screenshot). Users can query a food 1D ^1^H NMR spectrum that is binned and in text data format. A row of the text data comprises a chemical shift and an intensity delimited by a tab. The data should be 716 and 547 bins ranging from 0.5 to 9.0 and 0.5 to 7.0 ppm for D_2_O and CDCl_3_, respectively. The user should set a similarity threshold, within which FoodPro searches similar NMR spectra. The user should select the solvent, of which only D_2_O or CDCl_3_ can be currently selected. The user can optionally specify three of the special chemical shift regions that are ignored for further computation such as similarity pattern match or covariance spectra. The primary use of this function is ignoring large solvent peaks in the query. Finally, the user can initiate the search by pushing the Search button. The user can also load an example query.

The structure of the results is described below. A result comprises a table of similar NMR spectra as a result of similarity pattern matches to the database content (Figure 1c, upper screenshot), tasting (or hardness) (Figure 1c, center and lower screenshots), estimated tasting (or hardness) (Figure 1d, upper), and covariance and correlation spectra (Figure 1d, lower) displayed in that order. The similarity is computed by a sum of squares of differences (see Materials and Methods). It should be noted that the lower the similarity value, the more similar are the spectra. The table of similar spectra includes information such as the names of samples and similarity to the query. The table of tasting (or hardness) includes information such as tasting and evaluation of taste by panel volunteers with a Likert-type five (5: best to 1: worst) point scale (Figure 1c, tasting). The table of hardness includes experimental measurement data by a penetrometer (Figure 1c, hardness). The estimated scores (Figure 1d, upper) are shown as a weighted average of corresponding tastings and hardnesses registered in the database to hit similar database NMR spectra, with hit maximum and minimum as a vertical line. If complete matches exist (similarities to the query are 0), the direct average among the complete match is additionally displayed. Covariance and correlation NMR spectra between chemical shifts and tasting or hardness contain covariance values along chemical shift (Figure 1d, lower). It computes a covariance value between NMR bins and either tasting or hardness values (see Materials and Methods). These spectra estimate whether tastings or hardnesses relate to bin intensities. Positive (or negative) covariance or correlation means bin intensities increase (or decrease) when tasting or hardness increases. FoodPro could, therefore, link a user query to the registered 1753 tastings and 139 hardnesses and offer a newly developed statistical analysis method for them.

**Figure 2:**
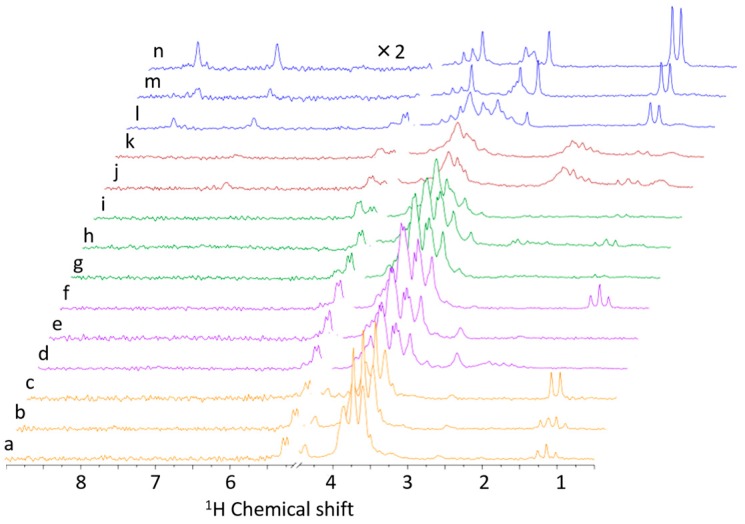
Typical experimental bench-top 60-MHz NMR spectra for processed foods deposited in FoodPro. (**a**) Milk with freshly added yogurt; (**b**) 6-h fermented milk with yogurt; (**c**) 20-h fermented milk with yogurt; (**d**) tomato fruit extract; (**e**) blueberry extract without fermentation; (**f**) 10-day fermented blueberry extract; (**g**) radish pickled in fermented rice bran; (**h**) cucumber pickled in fermented rice bran; (**i**) carrot pickled in fermented rice bran; (**j**) ramen soup with dried small sardines;, (**k**) ramen soup with pork bone and soy broth; (**l**) pickled bluefin tuna fermented in soy sauce; (**m**) raw sardine muscle extract; and (**n**) raw bluefin tuna extract.

Among the 367 total NMR spectra of foodstuffs deposited in the FoodPro database, typical stacked plots are shown in Figure 2 for D_2_O (Figure S2 in Supplementary Materials for CDCl_3_). From bottom to top, orange Figure 2a–c are a series of different durations of milk fermentation with yogurt, purple Figure 2d–f are fruits, green Figure 2g–i are vegetables, brown Figure 2j,k are ramen soups and blue Figure 2l–n are fish muscles. Overall, fish showed characteristic metabolites such as lactate (1.31 ppm, doublet), creatine (3.03 and 3.92 ppm), trimethylamine *N*-oxide (TMAO) (3.26 ppm), and histidine (7.28 and 7.89 ppm). In contrast, ramen soup exhibited characteristic signals of glutamic acid (2.1–2.3 ppm, multiplet), which may be an additive for enhancing umami taste. Other sugar-rich foodstuffs (vegetables, fruits, and yogurt) showed characteristic large peaks between 3.2 and 5.6 ppm, whereas milk with yogurt exhibited a different peak pattern of sugar signals due to a different main component (lactose). Furthermore, fermented products (ethanol triplet at 1.17 ppm in Figure 2a) contained lactate after 6 h in Figure 2b and were completely converted by 20 h in Figure 2c. Therefore, the bench-top NMR (60 MHz) can be used to monitor chemical reactions and compositional changes during food processing.

**Figure 3:**
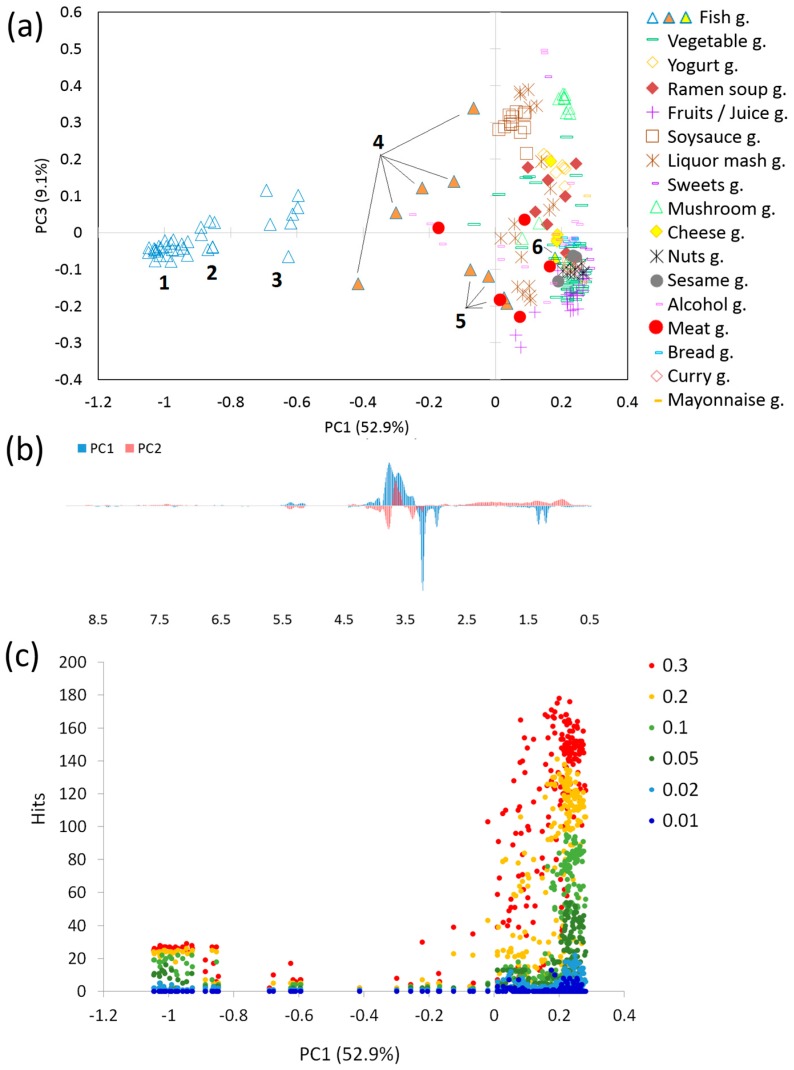
(**a**) Principal components analysis (PCA) score plot for PC1 and PC2 with 236 D_2_O spectra. Note that symbol names represent broad types of processed foods such as fermented or cooked (e.g., Fish g means a fish group). The fish group is classified into six groups (1–6, light blue triangles). These are further divided into three groups filled in white, orange, and yellow; (**b**) PCA loading plot for PC1 (blue) and PC2 (red); (**c**) Search sensitivity. Number of hits in the database vs. PC1 score are shown. Similarity thresholds are 0.01 (dark blue), 0.02 (light blue), 0.05 (dark green), 0.1 (light green), 0.2 (orange), and 0.3 (red). A point corresponds to a query NMR spectrum that has a PC1 score the same as that in Figure 3a.

Summary and search sensitivity of the core database of FoodPro is described below. Principal Components Analysis (PCA), which summarizes spectrum patterns, was performed for all D_2_O NMR spectra (Figure 3a,b). Currently, samples of fish and vegetables occupy 38% of the 236 D_2_O spectra. The PCA result clearly indicates that the fish and vegetable groups are separated along the PC1 axis (Figure 3a). This means that the groups of fish on the left side and vegetables on the right side have different spectrum patterns. At a greater level of detail and considering only the fish group, by simultaneously using the PC1 and PC2 axes, we could classify the entries into six smaller groups (Figure 3a, light blue triangles classified from 1 to 6 are filled in different colors). Marinated fish (groups 4 and 5, filled in orange) were differentiated from fish groups 1–3, which were raw, heated, or dried fish. This was due to the addition of sugars and other chemicals during the marinating process. A significant difference between the fish and not-fish groups was also detected (*p* = 2.4 × 10^−86^). The rest of the NMR spectra excluding fish and vegetables existed on the vegetable side in PC1. Within those spectra, alcoholic beverages, fruits, liquor mash, soy sauce, and nuts groups were major groups. These occupied 36.4% of the D_2_O NMR spectra. The fruits and nuts groups were separated from the group of soy sauce along the PC2. The alcoholic beverage and liquor mash groups were broadly distributed along PC2, similar to that of fish and vegetables. Contribution ratios in the PC1 and PC2 axes were 52.9% and 9.1%, respectively. This represented approximately 60% of the total variance of the NMR spectra. Loading plots indicated several specific peaks (Figure 3b). On the PC1 axis, positive peaks at 3.41, 3.65, 3.79, 5.21, and 5.38 ppm, and negative peaks at 1.25, 1.37, 3.02, 3.25, and 7.27 ppm, were observed. On the PC2 axis, positive peaks at 0.97, 1.42, 3.65, and 7.40 ppm, and negative peaks at 1.37, 3.02, 3.41, 3.79, 5.21, and 5.38 ppm, were observed. The peaks at 1.25 and 1.37 ppm were assumed to correspond to lactate, that at 3.02 ppm to creatine, that at 3.25 ppm to alanine, and that at 7.27 ppm to histidine. The peaks at around 3 and 5 ppm included signals that corresponded to sugars. These peaks were statistical averages and thus indicated slight chemical shift variability between samples. CDCl_3_ NMR spectra of the fish group also formed a specific region in the PC1–PC2 plane (Figure S3 in Supplementary Materials).

The sensitivity of similar spectrum detection during a query search of the D_2_O spectrum database was investigated (Figure 3c). We queried each of the NMR spectra in the database. This, therefore, meant an investigation of the distribution of distances between all the pairs of NMR spectra in the database. Similarity in FoodPro refers to the distance between query and database spectrum (see Materials and Methods). A lower similarity value indicates a more similar spectrum. We investigated search sensitivity, or numbers of hits, using different similarity thresholds from 0.01 to 0.3, as hits depend on a similarity threshold specified at the time of querying. It showed that spectra nearby the vegetable group, around +0.2 of PC1, exhibited large numbers of hits even when the threshold was 0.1 (Figure 3c, right side, light green) as the database spectra were crowded (Figure 3a, right side). Thus, the sensitivity for vegetable spectra was high. This could lead to unreliable estimated tastings or hardnesses. The hits for the vegetable spectra could be halved with a similarity threshold of 0.05, thereby offering an improvement. On the contrary, fish spectra, which were around −1 to 0.0 of PC1, exhibited adequate numbers of hits even the threshold was increased to 0.1 (Figure 3c, left side, light green) as the database spectra were not crowded. It, thus, had a high probability of estimating accurate statistics. A recommended value for the similarity threshold of 0.1 for fish spectra was concluded, which should be lowered as the position on the PC1 axis proceeds from left to right and be 0.05 at the position of the samples including the vegetable group. A search with a lower similarity threshold is recommended when the number of hits is too large.

**Figure 4:**
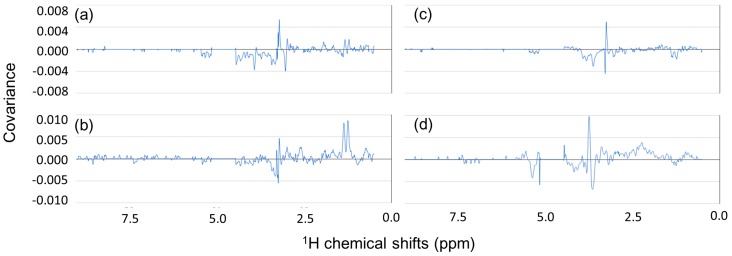
Covariance NMR spectra between chemical shifts and either tasting or hardness. (**a**) Covariance spectrum for tasting for a raw Japanese seabass; (**b**) Covariance spectrum for tasting for a steamed Japanese seabass; (**c**) Covariance spectrum for hardness for a raw Japanese seabass; (**d**) Covariance spectrum for tasting for a salmon roe.

Finally, we describe examples of covariance and correlation spectra for tasting and hardness. We concentrated on fish NMR spectra. Japanese seabasses are large, edible fishes commonly eaten in Japan and used in French-style cuisine. Querying the NMR spectrum for a raw Japanese seabass with a similarity threshold of 0.1 resulted in 18 hits, which comprised several types of fish, including other fish species, raw, steamed, boiled, and fried fish. Thirteen of these spectra had registered tastings in the database. Using them, FoodPro generated a covariance spectrum (Figure 4a); the spectrum is covariance vs. chemical shift. It means that positive (or negative) covariance indicates both higher (or lower) tastings and higher values of NMR bins. This spectrum was compared with that of a steamed Japanese seabass. It resulted in an increase of positive covariance at 1.31 ppm that is for lactate (Figure 4b). This means that a higher level of lactate corresponded, on average, to better tastes in the samples. A group of NMR spectra nearby the raw Japanese seabass sample in the database is a group located at the left-most position on the score plot (Figure 3a, fish group 1). The steamed Japanese seabass (−0.96 of PC1) was also in the group but located adjacent to the raw Japanese seabass (−1.03 of PC1). It resulted in slightly different hits, and hence, we could extract this difference in the covariance spectra.

The 18 hits searched with the raw Japanese seabass had 15 hits registered with hardnesses. They resulted in a covariance spectrum to hardness (Figure 4c). As a result, positive covariance to hardness at 3.26 ppm that is from TMAO, which is known to control osmotic pressure in fish [18,31]. This means that a higher level of TMAO corresponded to higher hardness in the fish group 1. We could also observe that this group had a higher level of TMAO than the fish group 3 (Figure 3a, around −0.6 of PC1, including bluefin tuna). Finally, we describe an example for salmon roe. Although salmon roe originates from fish, it was on the right side at +0.2 of PC1 (fish group 6). It was, in fact, closer to liquor mash or vegetables groups than the fish groups. Querying the salmon roe spectrum (0.05 of similarity threshold, nine hits of which five hits registered tastings) resulted in a covariance spectrum to tasting (Figure 4d). It was detected that a positive covariance to tasting at 3.47 ppm existed, which is for glucose. This is an example in which we could extract a peak of sugar with the covariance spectrum for tasting from multiple NMR spectra associated with processed and cooked foods.

Several analytical approaches tend to focus on a specific class of compounds or on a single compound important for evaluating foodstuff quality, nutritional properties, and sensorial characteristics. This type of analysis, also called “target analysis,” usually requires specific extractions and sample pretreatment. In contrast to this, NMR spectroscopy has gained an important role among the various analytical techniques used to analyze foodstuff owing to its “high-throughput” nature and high analytical precision [32]. Many applications have been reported in the literature showing the potential of an NMR-based approach to investigate the geographical origin, quality control, and processing of foodstuff [33,34,35]. In addition, different fish metabolites determining fish quality, taste, etc., may be simultaneously monitored and qualitatively described, making NMR spectroscopy a valid (and in many aspects, unique) tool to characterize the metabolic profile and properties of fishes [36,37]. A further advantage of NMR-based approaches is simpler and more direct sample preparation compared with that required for other analytical methods, which may involve derivatization or column chromatography. In this study, we demonstrated its practical use for food process characterization via bench-top NMR, which is a cost-effective instrument to evaluate foodstuffs [27]. In summary, we have developed FoodPro, a novel web-based tool that can search the food NMR spectrum database and compute estimated tastings, hardnesses, covariance, and correlation spectra. FoodPro is systematically designed for accepting versatile data other than tasting or hardness, and thus, can be easily extended to other indices. Combining the high-throughput nature of NMR with easy sample preparation, further accumulation of data and refinement of the web-tool function might contribute to the on-site characterization of foodstuff and processing in the future.

## 3. Materials and Methods

### 3.1. Accumulation of Experimental Data

A spectrum database in FoodPro was constructed by accumulating 367 newly measured 1D ^1^H NMR spectra of samples associated with food processing. These were acquired with a 60-MHz bench-top NMR (NMReady 60e, Nanalysis Corp., Calgary, AB, Canada) under measurement parameters with 1024 points, typically a spectrum width of 10 or 12 ppm, 64 to 10,240 scans, at 303 K, and using NMR references (0 ppm) of 4,4-dimethyl-4-silapentane-1-sulfonic acid (DSS), and tetramethylsilane (TMS) for D_2_O and CDCl_3_, respectively. The food samples were a broad group of processed or cooked fish, meat, vegetables, and beverages. For all the liquid samples except oils, the 1-mL samples were lyophilized or evaporated using a centrifugal evaporator, extracted in 600 μL of either D_2_O or CDCl_3_ for hydrophilic- or hydrophobic samples, respectively, and ultracentrifuged at 14,000 rpm for 5 min. The NMR spectra of the supernatants were then measured. The oils were directly dissolved in CDCl_3_. For all the solid samples with two-phase extraction, the lyophilized 80-mg samples were fragmented with an Automill machine at 15,000 rpm for 5 min, dissolved in 600 μL CDCl_3_, and ultrasonically homogenized for 15 min, then 600 μL of D_2_O was added, and the mixture was vortexed for a two-phase extraction. After letting the samples stand for 10 min, the NMR spectra of both supernatants in two phases, ultracentrifuged at 14,000 rpm for 5 min, were measured.

The intensities of all the registered D_2_O (CDCl_3_) binned spectral data were normalized. The D_2_O (CDCl_3_) binned data consisted of 716 (547) bins for the region from 0.5 to 9.0 (7.0) ppm, in which a bin was one of 1024 points in an NMR spectrum measured with a spectral width of 12 ppm. NMR spectra measured with 10 ppm widths were converted using Mnova NMR software 10.0 (Mestrelab Research, Santiago de Compostela, Spain) to adjust the bin sizes such that 1024 points corresponded to a width of 12 ppm.

Taste evaluations for the food samples were based on a five-point Likert-type scale and more than 30 panel volunteers performed these evaluations. The scale described the best as five and worst as one. Hardnesses for the food samples were measured with penetrometers (KM-5 and KM-1, Fujiwara Scientific Company Co., Ltd., Tokyo, Japan). Principal components analysis (PCA) was performed with prcomp function of R software 3.2.2 [38], as previously described [39,40].

### 3.2. Database Design

FoodPro was developed using MySQL, Apache, PHP, and JavaScript. FoodPro has six relational database tables, which are Spectrum, Tag, SpectrumTag, BSpectrum, Bin, and Feature tables. Spectrum table represents NMR spectra. Tag table represents tags that the developer can flexibly define. SpectrumTag table represents links between NMR spectra and tags to classify NMR spectra using tags. BSpectrum table represents binned NMR spectra. Bin table represents bins. Feature table represents feature vectors that are linked to binned NMR spectra. An element of a feature vector can be a double-precision number, integer number, or 32-length letters. Experimental tasting or hardness values were defined as integers and double-precision scalar values (one-dimensional vector) and were accumulated into Feature table. Multiple records of Feature table can be linked to the same binned NMR spectrum. For example, two different tasting evaluations by two panel volunteers to the same binned NMR spectrum were registered into Feature table as two distinct records wherein each of them had different tasting values.

### 3.3. Computation of Covariance and Correlation Spectra for Tasting and Hardness

A user query with an NMR spectrum to FoodPro generates covariance spectra along chemical shifts for tasting and hardness. A covariance is a covariance value between NMR bins and either tasting or hardness values. Firstly, FoodPro searches similar NMR spectra in the database using similarity $D=\sum_{i=1}^{M}{\left(q_{i}-Q_{i}\right)}^{2}$, where *D* is similarity, *Q_i_* or *q_i_* is the normalized intensity of the *i*-th bin in the user query or hit spectrum, respectively, and *M* is the number of bins. Within a spectrum pattern match, bins less than the maximum intensity divided by 75 are set to zero for noise filtering and divided by the sum of the squared bin intensities for normalization. Database NMR spectra that have similarity *D* less than or equal to a similarity threshold (specified at the time of querying) are hit as similar spectra to the user query. Let *N* or *P* be a number of hits of similar spectra to the user query or of tasting values retrieved from the corresponding hit spectra, respectively. The covariance spectrum between the user query and tasting is computed by $c_{i}=\frac{\sum_{j=1}^{P}q_{j}t_{j}}{P}-\bar{q}\cdot \bar{t}$. The computation for hardness is performed in a similar manner. In the expression, *c_i_* is a covariance for the *i*-th bin (*i* = 1, …, *M*), $\bar{q}$ is the average of the normalized intensity of the *i*-th bin corresponding to the *j*-th tasting (or hardness), *t_j_* and $\bar{t}$ are the *j*-th and average tasting (or hardness), respectively. In a similar way, the correlation spectrum between the user query and tasting (or hardness) is computed by the Pearson coefficient, $r_{i}=\frac{\sum_{j=1}^{P}\left(q_{j}-\bar{q}\right)\left(t_{j}-\bar{t}\right)}{\sqrt{\sum_{j=1}^{P}{\left(q_{j}-\bar{q}\right)}^{2}\sum_{j=1}^{P}{\left(t_{j}-\bar{t}\right)}^{2}}}$. The estimated tasting (or hardness) for the user query is a weighted average tasting (or hardness). The weighted average is computed by $A=\frac{\sum_{j=1}^{P}w_{j}\times t_{j}}{\sum_{j=1}^{P}w_{j}}$, where $w_{j}=\frac{1}{D_{j}+\bar{D}+1}$, and where $D_{j}$ and $\bar{D}$ are the *j*-th and average similarity, respectively. If there is one or more complete match (*D* = 0), an additional weighted average is computed by $A=\frac{\sum_{j=1}^{P\prime }t_{j}}{P\prime }$, where *P′* is the number of complete matches. Computations of covariance, correlation, and estimated scores were implemented in PHP and visualized in JavaScript with Line Chart and Candlestick Chart in Google Charts [41].

## Supplementary Materials

The following are available online at http://www.mdpi.com/2218-1989/6/4/36/s1, Figure S1: System and entity–relationship diagrams of FoodPro, Figure S2: Typical CDCl_3_ experimental NMR spectra deposited in FoodPro, Figure S3: (a) PCA score plot for PC1 and PC2 with CDCl_3_ spectra. Note that symbol names represent broad types of processed foods such as fermented or cooked (e.g., Fish g means a fish group.) (b) PCA loading plot for PC1 (blue) and PC2 (red).
