# Supplementary material for: FoodPro: A Web-Based Tool for Evaluating Covariance and Correlation NMR Spectra Associated with Food Processes
Source: Metabolites. 2016 Oct 19;6(4):36. doi: 10.3390/metabo6040036 (PMC5192442; doi:10.3390/metabo6040036)
Supplement: Supplementary file 1 [file metabolites-06-00036-s001.pdf]

Supplementary Materials: FoodPro: A Web-Based Tool for Evaluating Covariance and Correlation NMR Spectra Associated with Food Processes

Eisuke Chikayama, Ryo Yamashina, Keiko Komatsu, Yuuri Tsuboi, Kenji Sakata, Jun Kikuchi and Yasuyo Sekiyama

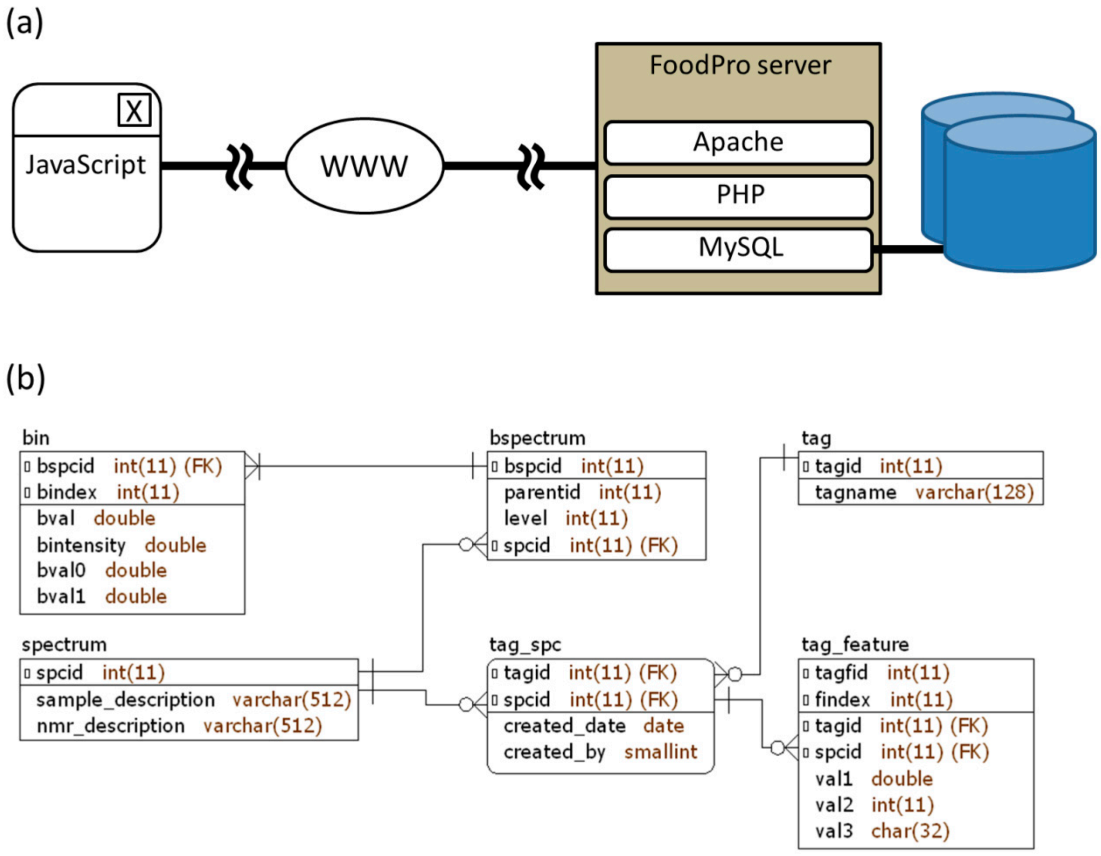

Figure S1. System and entity-relationship diagrams of FoodPro. (**a**) System diagram. FoodPro is accessible with a web browser. It connects a web server, Apache via the Internet; Apache executes PHP programs; PHP accesses MySQL database and retrieve data; PHP generate HTMLs embedding JavaScript codes; Apache returns the HTMLs to the browser; and finally the browser executes JavaScript and display FoodPro contents; (**b**) The entity-relationship diagram of FoodPro implemented in MySQL database. FoodPro has six relational database tables. Those are Spectrum (spectrum), Tag (tag), SpectrumTag (tag_spc), BSpectrum (bspectrum), Bin (bin), and Feature (tag_feature) tables.Primary keys are drawn upper sides in the rectangles. Field names and data types are drawnin black and brown, respectively. Foreign keys are labeled as FK.

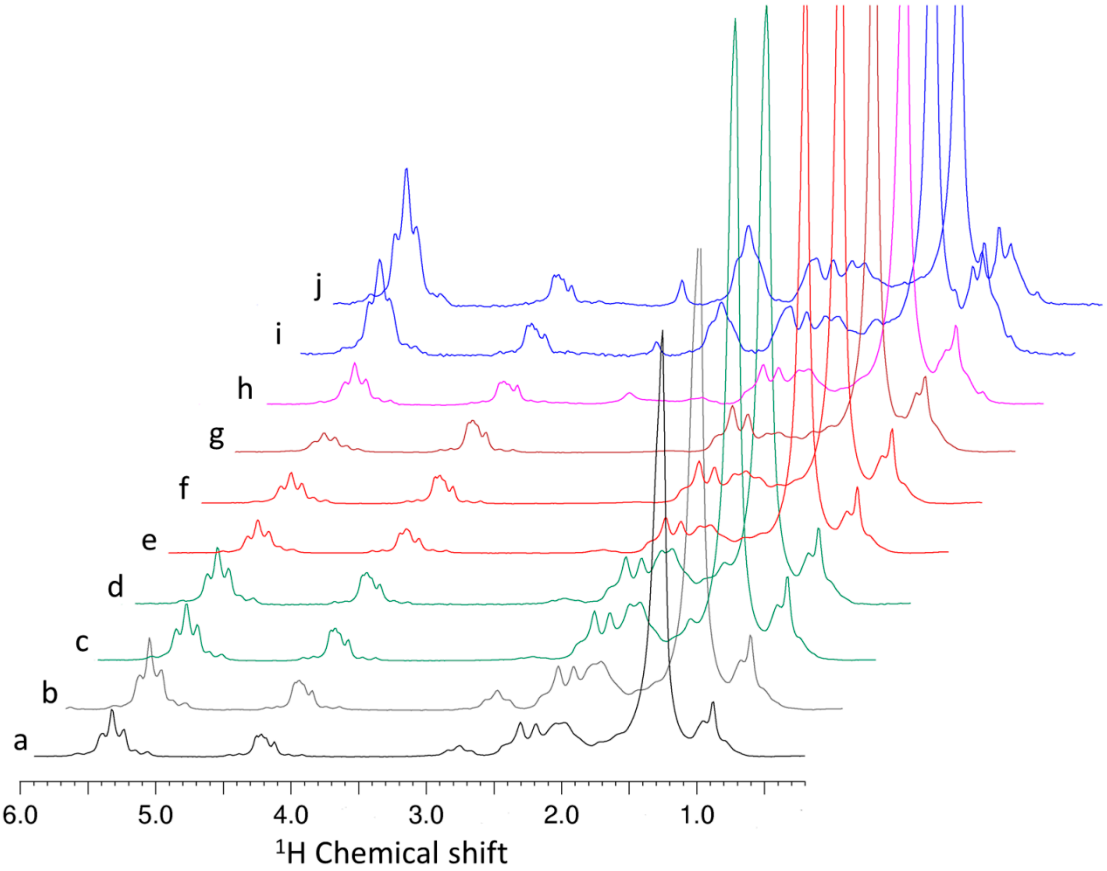

Figure S2. Typical $CDCl_3$ experimental NMR spectra deposited in FoodPro. (**a**) Peanuts; (**b**) sesame; (**c**) avocado; (**d**) extra-virgin olive oil; (**e**) grilled pork; (**f**) beef tallow; (**g**) coconut oil; (**h**) egg; (**i**) salmon roe; (**j**) raw grouper.

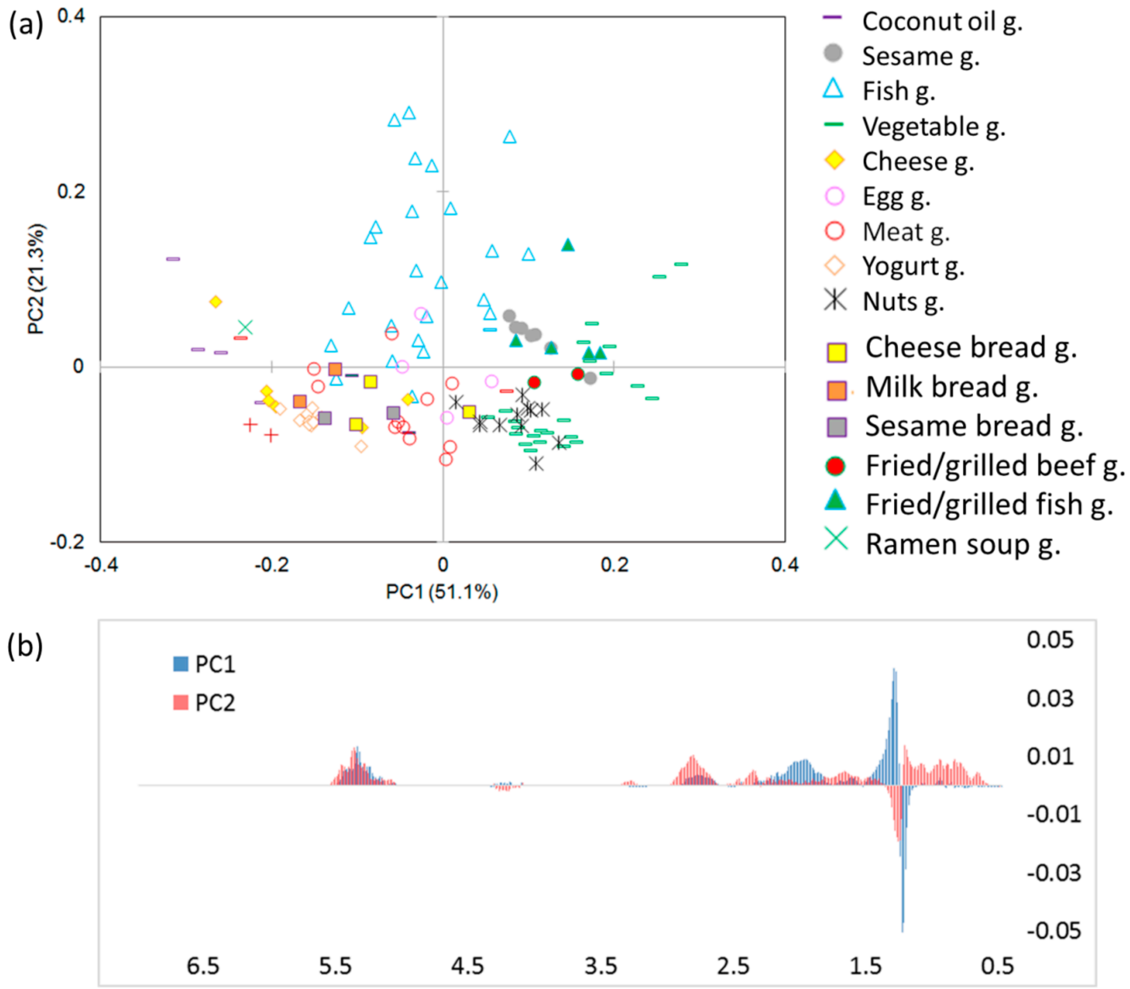

Figure S3. (**a**) PCA score plot for PC1 and PC2with $CDCl_3$ spectra. Note that symbol names represent broad types of processed foods such as fermented or cooked (e.g., Fish g means a fish group.); (**b**) PCA loading plot for PC1 (blue) and PC2 (red).
